# Class-agnostic annotation of small RNAs balances sensitivity and specificity in diverse organisms

**DOI:** 10.1016/j.csbj.2025.05.045

**Published:** 2025-05-27

**Authors:** Nathan R. Johnson, Fabian Gonzalez-Toro, Barbara Bernal Gomez

**Affiliations:** a Centro de Genómica y Bioinformática, Facultad de Ciencias, Ingeniería y Tecnología, Universidad Mayor, Chile; b Millennium Science Initiative - Millennium Institute for Integrative Biology (iBio), Chile

**Keywords:** Eukaryotes, Total small RNAs, Non-model organisms, TinyRNAs, MiRNAs, SiRNAs, PiRNAs

## Abstract

Small RNAs (sRNAs) are important regulatory elements in eukaryotic organisms and comprise the functional elements of RNA interference. Numerous classes of sRNAs have been annotated, however they vary greatly in their ease of annotation and compatibility with most annotators. Significant challenges exist for the annotation process, including variation in sRNA library quality, alignment depth, and poorly defined loci, collectively making this process difficult. Additionally, few annotators are fully agnostic to sRNA classes and may struggle identifying loci in less explored organisms. To address these problems, we present the annotation tool YASMA-tradeoff (YTO), which is specifically suited to finding reliable thresholds for locus annotation which balance sensitivity with specificity. We compared YTO with other annotators, we show that it and other pipelines based on coverage-normalization methods have great advantages, balancing many metrics to produce a more reproducible annotation. We also demonstrate that YTO produces more contiguous and representative loci, through the aggressive merging of similar expressed regions. Finally, we also show that the tool produces much more descriptive locus dimensions, a major advantage in species where sRNAs may be distinct or unique. Overall, we demonstrate substantial improvements in annotation accuracy, reproducibility, and description, particularly in non-model organisms and less-explored clades.

## Graphical Abstract

**Figure:**
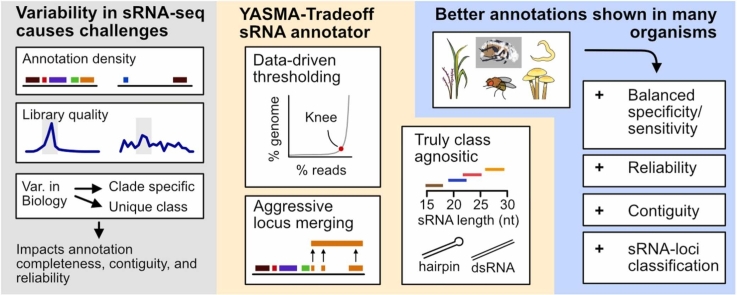

## 1 Introduction

Small RNAs (sRNAs) are the functional elements behind RNA interference (RNAi), a system of genetic regulation ubiquitous among eukaryotes. The core-machinery responsible for sRNAs are ancient and widely conserved [33], [45]. Processing follows the same general scheme: double-stranded RNA is processed by a type-III RNAse (Dicer or Dicer-like, here referred to collectively as Dicer) and loaded into an Argonaute family protein which carries-out specific RNAi functions [37], [46]. Some sRNAs have been shown to be processed independent of Dicer, such as piRNAs [38] and some classes of sRNA in fungi [24]. Within eukaryotic kingdoms, sRNA classes are mostly well conserved in processing and function, with some classes even found across all eukaryotes [10], [24], [3]. Specific loci are also widely conserved, as seen with microRNAs (miRNAs) where orthologous loci are found in diverse organisms [7]. Despite these similarities, there are also many differences in the sRNA content and classes between various organisms and clades. This highlights the complexity and adaptability of sRNA pathways across species.

Annotation is the process of sRNA expression in to genetic units, providing a foundation for understanding the biology of sRNAs. This usually involves clustering sRNAs into genomic loci, classification based on characteristics, and sometimes identification of homology and function. This process is used to help understand the sRNAs that exist in an organism and is often the first step to understanding their function. Unlike in RNA-seq, where sequences come from the *in vitro* fragmentation of larger transcripts, sRNA-genes are naturally fragmented by Dicer [13]. Moreover, sRNAs are short and sometimes sparsely aligned, making contig construction without a reference genome usually impractical. A prime example of this are miRNAs, which by definition predominantly produce 2 distinct sRNAs from different arms of their precursor hairpin [2]. These precursors are sometimes detectable via directed amplification efforts in animals but are rarely, if-ever, found in plants [3]. Other types of sRNA loci present even greater challenges due to their diverse precursors and less defined processing mechanisms. For instance, RNA-dependent RNA polymerase-derived siRNA loci in plants are shown to be produced by an assortment of intermediate-sized precursors, which are the products of many transcriptional initiations [4]. While other mechanisms are known [34], the full details of transcription remain largely unknown for many sRNA-classes, particularly in less studied clades such as fungi.

As annotation is usually the first step in sRNA analysis, it follows that many annotators have been published, with more than 5,500 collective citations in PubMed (Fig. S1). However, these tools vary considerably in terms of annotation quality and focus. Most tools are exclusively focused on miRNAs, including the commonly used tools of the miRDeep-family [43], [9] and miRDeepfinder [40]. This preference is likely due to the relatively well-defined nature of miRNA loci [2] and their clear specific phenotypes [36]. In contrast, less defined classes such as many siRNAs tend to be more challenging to assess and are often ignored by annotators (Fig. S1). Nevertheless, these classes are crucial to understanding sRNAs in organisms that produce major portions of siRNAs (e.g. plants) [29], piRNAs (e.g. some animals) [17], or those with hairpin RNAs that are less clearly defined (e.g. fungi) [18], [24]. Some tools do not perform *de novo* annotations, relying solely on previously published loci for their analysis (Fig. S1), usually limiting the scope of these tools to well-defined sRNA classes [11]. Other tools use genome-alignment-free approaches, also narrowing the range of loci that can be discovered [39], [44]. This leaves a vanishingly small set of annotators which might be capable of defining sRNAs in organisms with little or no definition of their sRNA classes [1], [25].

Major hurdles persist in small RNA annotation. Library quality has a significant impact, often leading problematic annotations [28]. Variation in sRNA classes across eukaryotes introduces challenges, as most existing tools are primarily tested in a limited range of species with well-defined locus parameters. Critically, virtually no tools are designed for *de novo* sRNA annotation outside of plants and animals, leaving a significant gap in understanding less-characterized organisms. Natural variation in terms of signal and noise between organisms and libraries further complicates annotation efforts, particularly for species that may have lost some or all functional RNAi machinery [8]. Together, these factors create significant challenges for defining locus margins and preventing false annotations. These problems are further exacerbated when performing large-scale analyses over many samples and projects, as the risk of exceptional and erroneous samples increases. To address these challenges, this study introduces a data-driven approach that combines threshold optimization and aggressive merging to enhance annotation accuracy. By comparing our methodologies with widely used tools, we demonstrate clear distinctions in how sRNAs are annotated. We show our approach offers robust solutions for producing more consistent annotations across a broader range of organisms, paving the way for improved sRNA characterization in previously underexplored clades.

## 2 Results

### 2.1 Small RNA data are variable between diverse species

**Fig. 1:**
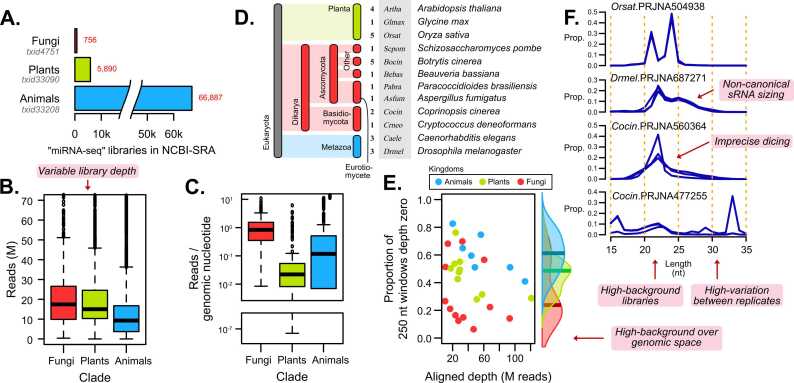
Challenges with sRNA annotation at large. A) Raw counts of “miRNA-seq” libraries in NCBI-SRA. B) Distribution of raw read-counts. C) Global spatial density over the genome. D) Selection of species included in the test-set of sRNA-seq bioprojects. E) Proportion of zero-depth 250 n nucleotides bins plotted against total aligned depth. Points represent Bioprojects and are colored by animals (red), plants (green), and fungi (blue). F) Size-profiles of example alignments of several bioprojects, with replicate libraries shown as overplotted lines.

Since the advent of high-throughput sequencing, many species have been studied using sRNA-seq. Searching the NCBI-SRA for “miRNA-seq” libraries reveals tens of thousands of entries (Fig. 1A), predominantly from animals, but with a growing representation of plants and fungi (Fig. S2). However, these libraries are not all equivalent. For instance, library depth varies considerably, with fungi and plants generally exhibiting higher read depths than animals (Fig. 1B), possibly due to higher-depth from more recent sequencing efforts. Fungi typically have much smaller genomes than plants and animals, leading to deeper library coverage over genomic space in fungi (10–100x) compared to the other clades (Fig. 1C). While this high functional depth (around median 1 read per genomic nucleotide) can enhance resolution, it also poses challenges for annotation, particularly for tools optimized for sparsely distributed alignments like those observed in plants.

To explore this relationship in greater detail, we selected a representative set of publicly available libraries from bioprojects in the NCBI-SRA, including plant (10), animal (6), and fungal (12) projects from a range of species (Fig. 1D). These focused on getting multiple projects for several organisms with variation in sequencing depth, requiring only that they maintained a size-profile that had a distinct sRNA peak (Fig. S3). We aligned wild-type control replicates to their reference genomes and assessed the alignment coverage. By segmenting the genome into 250 nucleotide bins, we observed that fungi have a low proportion of the genome with no aligned reads (median less than 20 %) (Fig. 1E). When comparing aligned depth with this zero-proportion, we identified a trend: deeper libraries generally exhibit less zero-depth-space (Fig. 1E), suggesting that both sequencing depth and genome size contribute to the dense alignments observed in fungi. This ubiquity may result from high noise in regions that are not producing sRNAs or other forms of transcription.

Looking at some selected size profiles of aligned sRNAs across different species, we observe several challenges associated with real data. For instance, *Oryza sativa* (*Orsat*) represents a characteristic clean profile from a plant, with sharp peaks at 21 and 24 nucleotide (Fig. 1F). In contrast, *Drosophila melanogaster* (*Drmel*) exhibits two types of non-coding RNA: a 22 nucleotide peak and a broader 25–28 nucleotide peak, the second of which might not be found by annotators by default. Broader or more diffuse peaks found in some libraries may be an indication of imprecise dicing or other mechanisms specific to an organism (Fig. 1F). Library quality presents a major hurdle, as low-quality libraries can severely distort the results of an analysis, such as seen in *Coprinopsis cinerea* (*Cocin*) project PRJNA477255 (Fig. 1F). Additionally, library depth plays a crucial role in annotation, with large variations in raw reads (Fig. 1B) and genomic background (Fig. 1E) further complicating the density of reads in the alignment. Finally, discrepancies in library size and profile among replicates justifies careful normalization prior to annotation to prevent disproportionate influence from deep or problematic libraries (Fig. 1B, F).

### 2.2 Annotating sRNAs balancing sensitivity and specificity

Many tools are available for small RNA annotation, varying widely in their scope and strategy (Fig. S1). Here, we focus on annotating sRNAs agnostic of class definitions, leading us to consider ShortStack [19] due to its wide usage and command-line interface. Preliminary work with ShortStack version 3 (SS3) revealed difficulty with high-density alignments, such as those observed in fungi (Fig. 1E). This issue manifested in several locus phenotypes: *over-merging*, where apparently different loci get clustered into a single locus; *under-merging*, where a larger locus is split into sequential loci; and *over-annotation* - where regions with trivially low expression are identified as loci. We reasoned that these problems stemmed from the way SS3 defines locus margins and proposed that a more refined approach to locus identification, expansion, and merging of loci could result in more-realistic alignments.

**Fig. 2:**
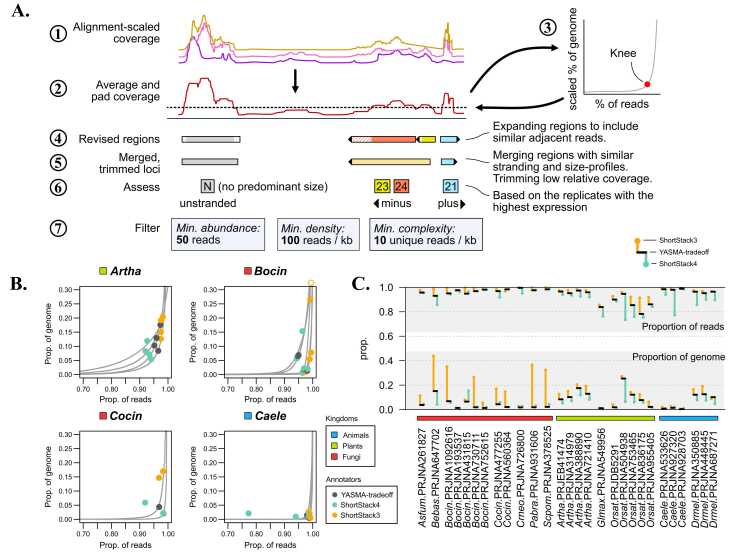
Thresholding sensitivity to avoid over-annotation. A) Overview of the YTO annotation method, with numbers indicating the progression of steps. B) Example tradeoff curves measured by YTO for several species (grey), with points showing the selected weighted tradeoff value. Hollow circle indicates a point outside the graphing window. C) The overall annotation rates for all three tools. Proportion of genome included (bottom) and proportion of reads (top) included in annotations are shown, with YTO (black bar), ShortStack3 (point connected to bar by line, orange), and ShortStack4 (same, aquamarine). Alignment depths (M reads) are shown below. Colors represent kingdoms: plants (light green), fungi (bright red), and animals (light blue).

This motivated the development of our own tool to perform annotations where we would address challenges related to alignment density, variation in library size and profile, as well as non-canonical small RNA sizes that may exist in less explored clades. Our tool, named YASMA-tradeoff (YTO), follows the procedure outlined in Fig. 2A (detailed in Methods and Materials). This first involves normalizing coverage for each library individually and balancing influence among replicates. The threshold-depth for annotation is determined using a knee-finding algorithm [35] which balances between annotating the *least genomic space* and annotating the *most reads*. This threshold is then used to define regions which are subsequently merged based on proximity and similarity in size-profile, stranding, and expression patterns. Basic filters are employed to make sure that regions have a minimum complexity and abundance to accurately assess their contents (Fig. 2A). These filters favor reducing false discovery at the cost of some sensitivity, contending that greater sequencing depth is required to annotate the filtered loci. YTO also allows for library-specific annotation within a larger sRNA project alignment, simplifying the process of alignment, annotation, and analysis for projects with multiple treatments, not all of which should be used for annotation (e.g. pleiotropic mutants).

Defining the threshold is one of the critical aspects of annotation by expression. ShortStack version 4 (SS4) is a revision of the ShortStack methodology which adopts normalized-coverage-based cutoff for locus identification, set at 1 read-per-million (RPM). Setting a simple global cutoff in terms of RPM is a simple approach which improves upon the prior strategy, yet this may result in some biases depending on the shape of the alignment.

Comparing this with YTO, we observe similar performance in many organisms, although in some cases SS3 and SS4 produce outlier results (Fig. 2B). SS3 tends to annotate much larger portions of the genome across all our test organisms (Fig. 2C). This trend is even more pronounced in fungi, where in some species it annotates as much as 40 % of the genome as sRNA-loci, echoing the preliminary results. Overall, SS4 and YTO perform similarly (Fig. 2C, S5, S6). The key exceptions are that YTO annotates more sRNAs in plants and animals whereas SS4 can tend to be more conservative (Fig. 2B). A notable point is *Orsat* project PRJNA504938 (Fig. 2C), which has very high depth and a large genome. YTO and SS3 both define much more of the genome as sRNA-loci, suggesting that this genome produces many loci which are only detectable with high-sequencing depth and might be lost by a strict RPM threshold.

### 2.3 Locus dimensions and contiguity vary between methods

Contiguity of loci is important for defining discrete, genomic units of sRNA expression. We reasoned that closely adjacent region (<500 nucleotides) with similar expression and read profile were likely to be transcriptionally linked.

**Fig. 3:**
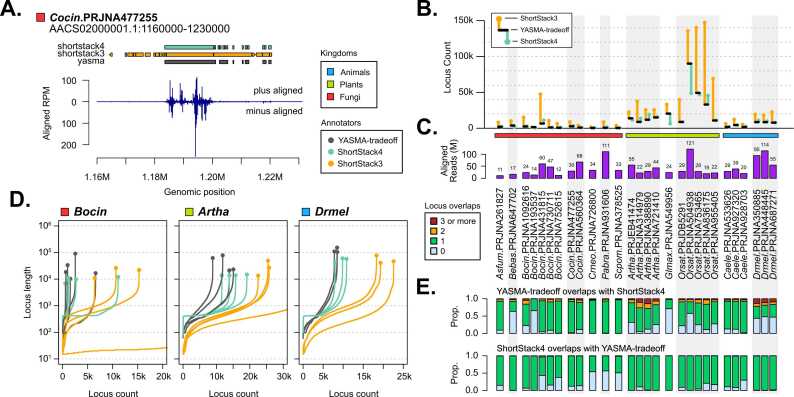
Variable locus contiguity and sensitivity. A) An example of a genomic window, showing annotations for ShortStack3 (SS3, orange), ShortStack4 (SS4, light green) and YASMA-tradeoff (YTO, black). Values are shown as coverage in reads-per-million, separating plus-strand (top) and minus-strand (bottom) alignments. B) Annotation dimensions for tested bioprojects, showing the overall locus count and medians for inter-locus gaps and locus lengths. YTO (black bar), SS3 (point connected to bar by line, orange), and SS4 (same, aquamarine). C) Alignment depths (M reads) for each bioproject. D) Locus length plotted against cumulative count of loci for three organisms. Line colors represent the tool as above. Terminal points on lines indicate the largest locus size. E) Counts of the number of SS4 loci which overlap with each YTO locus (top) and the inverse (bottom). Colors indicate zero (light blue), one (light green), two (orange), and three or more (red) overlapping loci.

To assess how loci compare between annotators, we next focused on their attributes. Looking at an example locus in *Cocin* project PRJNA477255 (Fig. 3A), we see YTO and SS4 are more exclusive in their identification of loci. Here, SS3 appears to exhibit under-merging/over-annotation, with many directly sequential loci. Merging from YTO can be seen with several of the loci, where one YTO locus is found in two or more SS4 loci. This can also be seen by looking at the raw locus counts for all three methods. YTO and SS4 tend to produce similar counts, whereas SS3 consistently identifies a dramatically higher number of loci (Fig. 3B, S7). This pattern is not limited to fungi but is observed across virtually all organisms tested. Aligned read-depth for these bioprojects is are not strongly correlated to locus counts (Fig. 3C). The exception is in *Orsat* project PRJNA504938, where again YTO and SS3 find many more loci than SS4, a possible sign of many unannotated loci due to low normalized depth.

By plotting the cumulative locus count versus locus length, we find that YTO tends to find longer loci than other methods (Fig. 3D). In *Arabidopsis thaliana* (Artha) and *Drmel*, the tools behave predictably between bioprojects in terms of count and length profile (Fig. 3D). Projects from the fungi *Botrytis cinerea* (*Bocin*) present greater challenges, exhibiting higher variance in locus count for SS3 and SS4 annotations and more stable performance for YTO. Size-profiles for these annotations show SS4 imposes a hard minimum size for loci (around 500 nt), whereas the other tools allow for smaller loci (Fig. 3D). In general, SS3 finds many more small loci (< 500 nucleotides) than the other methods, supporting the phenotype seen in Fig. 3A.

One possible explanation for the identification of larger and fewer loci with YTO is that it merges multiple distinct regions analogous to those in SS3 and SS4. To explore this, we examined the intersections of loci between tools. This showed that indeed many loci annotated in YTO overlapped with two or more loci identified in the other tools (Fig. 3D, S8). This is particularly distinct in SS3, where as much as 40 % of YTO loci contain 2 or more SS3 annotations (Fig. S8). Looking at the gaps between loci, we confirm that YTO is producing much less closely adjacent loci than in SS3 and to a lesser degree SS4 (Fig. S9). For all the tools we find a number of loci in bioprojects which are not found in others. Looking at the expression of these loci in SS3 and SS4, we find most make up less than 2 % of aligned reads in total, indicating many loci with very low expression.

### 2.4 Improved on reducibility in annotations with higher sensitivity

**Fig. 4:**
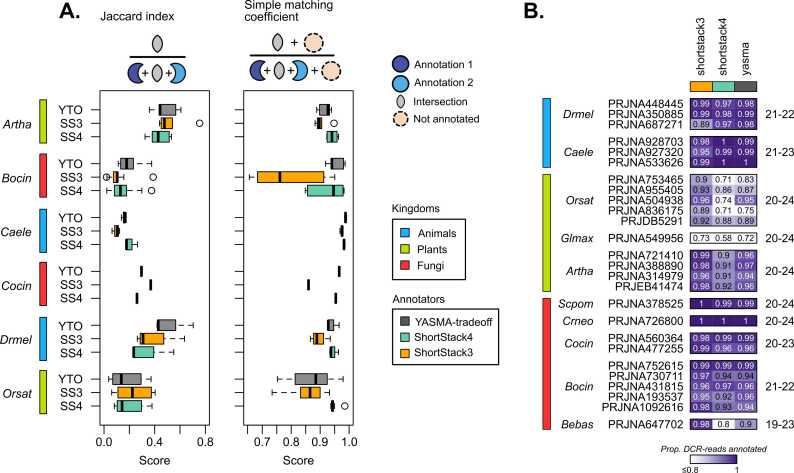
Evaluating annotation reproducibility and sensitivity. A) Pairwise annotation similarity between bioprojects in each organism. Left - Jaccard index, right - simple matching coefficient. B) Annotation sensitivity of Dicer-derived reads. Dicer-derived size-profiles are shown to the right of the heatmap. Color scale indicates the proportion of Dicer-sized reads annotated.

As high-quality annotations are the obvious aim of all these tools, we next tried to assess the confidence of these annotations. Limited validated-data on sRNA-annotations are available in any organism and are restricted to predominantly miRNAs [20]. We decided that reproducibility of annotations between projects could be a good proxy for quality, as loci that are consistently found between laboratories and experiments are more likely to be real sites of expression. For those organisms where we tested multiple projects, we performed pairwise intersections of the annotations to measure their similarity. Looking at the overlap of annotated space (Fig. 4A, Jaccard index), we find that some organisms have highly dissimilar projects and have generally low overlap with all of the tools. We considered that identifying non-annotated regions is also important, especially in high-noise libraries where we might experience over-annotation. For this, we utilized symmetrical comparisons (Fig. 4A, simple matching coefficient), which also evaluates the unannotated portions of the genome. Here, we find that YTO and SS4 tend to perform the best, showing that the specificity of these tools yields higher reliability. This is particularly clear in *Bocin* projects where SS3 shows very low reliability.

We then explored the sensitivity of each of these approaches. Using known Dicer profiles (plants and animals) as well as the size-profiles of our sRNA libraries (fungi) (Fig. S3), we built a list of sizes likely to be sRNAs in our samples (Fig. 4B - right). By testing the proportion of reads from these size-profiles that are in annotations, we can measure the sensitivity of Dicer-derived read annotation, considering unannotated reads to be false-positives (Fig. 4B). Here, we show that most tools behave similarly in animals and some fungi. In plants, SS4 shows lower sensitivity than the others, most dramatically in those with larger genomes (*Orsat*, 389 kB; *Glycine max*, 978 kB). Overall, this points to a higher read-sensitivity in YTO with little cost in reproducibility.

### 2.5 Capturing imprecisely processed and non-canonical loci

An ongoing concern for annotation in fungi and other yet-unexplored organisms, is that loci may appear different than those described in well-studied organisms. The sizes of constituent sRNAs within a locus provide valuable insights into its function, as specific sizes are known to be associated with biological roles and certain machinery/processes. ShortStack infers locus processing using a value called “DicerCall”, which identifies loci where a large proportion of reads fall within a range known to be Dicer-related. Those that pass this test are “called” based on their most abundant size. This is a very powerful tool for inferring a biological role of a locus. However, it also suffers in samples that fall outside of canonical ranges or have higher noise - possibly due to imprecise processing. YTO adopts a less strict approach, focusing on classifying loci based on the most common contiguous sizes, allowing up to a range of 3 major sRNA sizes for a locus. This strategy enhances sensitivity for loci which might have dispersed, but still contiguous sizes, or for loci that are likely not precisely processed by a Dicer protein (e.g. piRNAs). This is particularly important for exploring sRNA loci in less-characterized organisms, where clearly delineated processing patterns may not exist.

**Fig. 5:**
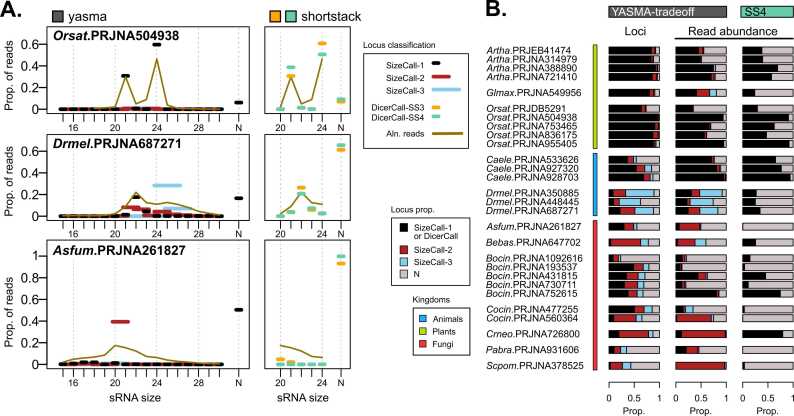
Locus description is methodology-dependent. A) Size-profiles of small RNA libraries in several bioprojects. Brown line shows the proportion of reads for each length of read. Lines with circles indicate proportion of all reads that fall into size-categorized loci: “sizecall” for YASMA-tradeoff (YTO) with locus widths of 1 (black), 2 (dark red), or 3 nt (sky blue), “DicerCall” for shortstack3 (SS3) and shortstack4 (SS4). B) Annotations from YTO and SS4 (aquamarine), showing the proportion of loci count and abundance for sizecalls-1, −2, and −3 (as described above), and “N” loci (grey).

Looking at three example profiles, we can compare how these methods perform by examining the abundance of reads categorized into each size-group (Fig. 5A). In *Orsat*, the results are quite similar between “sizecall” and “DicerCall”, with nearly all reads derived from loci called as 21- and 24-nucleotide loci. In *Drmel*, 21-nucleotide loci are annotated similarly by both approaches, but reads derived from larger loci within the piRNA range are grouped as “N” in ShortStack. In contrast, YTO classifies these as predominantly 24_25_26 and 25_26_27 loci, greatly reducing the “N” unknown loci. For *Aspergillus fumigatus* (*Asfum*), we see a highly dispersed sRNA profile centered around 20-nt. ShortStack fails to identify virtually any loci with a defined size-profile, with > 95 % of reads categorized as “N”. YTO, however, identifies many sRNAs as 20_21 loci, reducing the number of “N”-loci derived reads to approximately 50 %.

Looking at the proportions of the “sizecalls” among all test libraries, we can see several that benefit from this approach. Organisms such as *Bebas* (*Beauveria bassiana), Cocin*, *Crneo* (*Cryptococcus deneoformans*), *Scpom* (*Schizosaccharomyces pombe*), and *Drmel* all display large numbers of loci with broader size distributions (Fig. 5B). Furthermore, for several of these organisms, these larger sized loci represent the majority of annotated sRNA abundance (Fig. 5B), underscoring the limitations of precise-locus-only analyses and highlighting the value of YTO's flexible approach.

## 3 Discussion

### 3.1 No gold standard for sRNA annotation

This study has been focused on comparing the resulting annotations from YTO and ShortStack tools. A classic approach to this would be binary classification, assessing the annotations in the context of which loci are real/not-real and annotated/not-found. Unfortunately, there remains no gold standard annotation or annotative approach for sRNA-producing loci. Several annotations do exist in databases [21] and repositories of annotations [29], [5], but, using these as a standard to compare annotations is inappropriate, as their own annotations are subject to the methodology-used (Fig. S1). We instead focus on indicators of quality based on comparing our annotations and general characteristics. We have proxies for some key metrics: proportion of reads annotated is similar to sensitivity and proportion of genome annotated is similar to specificity (Fig. 2). Additionally, we compared annotation reliability (Fig. 4), Dicer-derived-read sensitivity (Fig. 4), and annotation over/under-merging (Fig. 3, S9).

**Fig. 6:**
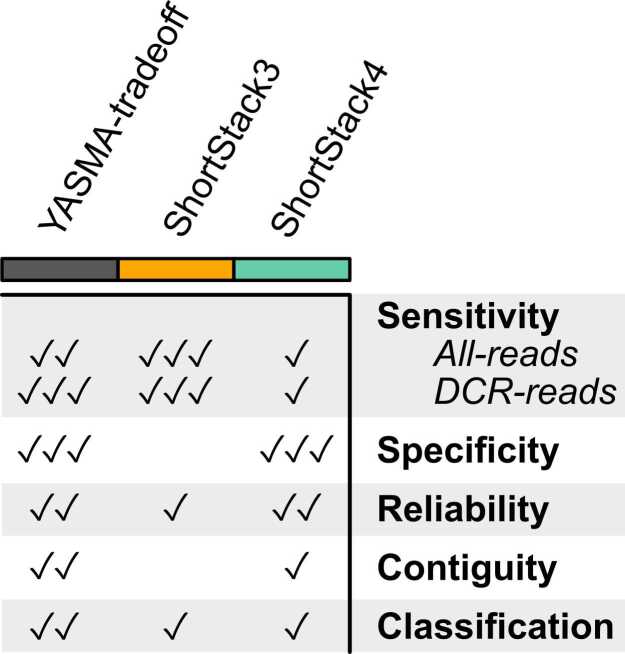
YASMA-tradeoff balances metrics of annotation quality. General summary of annotative quality across several metrics. Relative scores are shown on a scale of 0–3 check marks.

Among these metrics, different approaches have different strengths, outlined generally in Fig. 6. SS3 shows extreme sensitivity, annotating the highest percentages of reads. This comes at the cost of annotating vastly more loci than the other tools, many of which are directly adjacent to other loci and small. It also struggles in some organisms, namely fungi, annotating large regions of the genome as single loci. SS4 is more conservative, producing lower counts of more isolated loci, which are in-turn more reliable. This has the cost of lower sensitivity than the other methods, particularly in plants. YTO manages to balance the benefits and problems of these approaches. It retains high read-annotation sensitivity, matches SS4 in terms of reliability, and produces the most-contiguous loci of any tool.

### 3.2 Classifying loci is key for understanding role

Noise plays a major factor in sRNA annotation with high-depth libraries, as separating sRNA loci from the noise can be a challenge. RNAs in the size-range of sRNAs can be produced as the natural product of RNA-degradation [15], producing native noise in all samples. Deciphering whether an expressed region is derived from directed processing, often by a Dicer protein, is key to identifying loci that may have a regulatory role. DCRs produce specific-sized sRNAs [13], whereas degradation produces RNAs with irregular or random sizes. Similarly, specific AGO proteins preferentially bind to particular sRNA lengths [30], directly linking the size of an sRNA locus with its biological function. Here, ShortStack takes a conservative approach, applying a standard more-suited for the precision found in plants. In contrast, YTO adopts a broader approach, allowing for many more loci to be categorized and assessed based on their observed characteristics. While this approach weakens the ability to determine whether locus is Dicer-derived, it opens up possibilities for analyzing other small RNAs, particularly in cases where DCRs act differently, or cryptic dicing might occur [24], [42]. In these cases, strong genetic evidence is required to confirm which Dicer performs specific processing, meaning that any predictive tool is likely not sufficient.

### 3.3 Aggressive merging allows for more realistic loci

Locus merging is an important aspect of sRNA annotation. Transcription of sRNAs varies between classes, with some derived from processed RNA Polymerase II transcripts (miRNAs, tasiRNAs, phasiRNAs) [46] and others likely from many one-to-one transcription events (siRNAs) [4]. These make annotation contiguity challenging, as there can be large gaps in coverage between regions that are likely transcriptionally linked. YTO makes a strong effort to merge highly similar loci at long distances (500 nucleotides), forming much more contiguous annotations. This approach also impacts downstream techniques, as inflating one locus to multiples can affect the sensitivity of techniques like differential expression analysis.

Faulty annotations represent a major challenge for biological interpretation. Loci which are arbitrarily small, large, or divided can greatly alter the interpretation of what they are and what they do. This is evident in miRNA, where low-confidence annotations [2], [20] have influenced many subsequent publications. Annotators that produce higher rates of false annotations for sRNA loci run the risk of propagating false interpretations of classes, frequency of loci, and ultimately function of a given sRNA. By imposing stricter standards as YTO and SS4 show, this greatly reduces these risks.

### 3.4 Reliable methodologies are essential for new frontiers

With the rapid expansion of sequencing technology and data, an increasing number of projects aim to uncover meta-level trends within the genomes of organisms. This also includes the field of sRNAs, where numerous repositories of data have been compiled, including kingdom-wide studies [29], sRNA class-wide datasets [12], and even pan-target analyses [16], [27]. All these approaches leverage consistent methodologies for large-scale analyses, allowing for powerful comparisons across conditions, species, classes of sRNA, and more. We also show that YTO is well-suited to many classes of sRNAs, including those with non-canonical sRNA sizing, pushing back on these barriers. Other possible applications of this tool could be with other types of non-coding RNA, including those derived from other RNA sources (tRNA, rRNA) [32], [6] or alternative pathways (bacterial sRNAs) [26]. Further extending this approach with high-throughput methods of identifying targeting and function [14], [41] promises to link these annotations to their biological role.

Considering the large quantity of publicly available sequencing data, repository approaches linked to reliable methodologies show great promise [29]. We can leverage replication within a project and between projects, with the idea that consistent annotations are inherently more robust and likely to be real. This also opens the door for evolutionary studies, looking for conservation of locus type and specific loci on a clade-wide scale. This is particularly relevant for underexplored clades, where few works have shown signs of conservation [18] and a lack of common annotative approach have proved to be a serious hurdle.

## 4 Conclusion

Here, we present a comparison of how different approaches perform in sRNA-annotation, demonstrating that coverage-normalized methods like SS4 and YTO build much more consistent annotations. YTO uses threshold identification and merging to annotate a maximal amount of reads while retaining specificity. YTO’s merging constructs highly contiguous loci with more sensitive descriptions of size-profiles, enabling a broad identification of sRNAs. These annotators effectively address major challenges in terms of clade-wide analyses, which involve significant variation in sRNA libraries (quality, size, depth), genomes (sRNA types, expression, sRNA machinery), and functions. Here we show an important advancement in methodologies addressing sRNA annotation, allowing for greater insights into what sRNAs are made in organisms spanning the whole tree of life.

## 5 Methods and materials

### 5.1 Assessing and gathering public data

To find global metrics of public sRNA-seq data, we searched the NCBI using edirect tools. Our term looked for libraries matching the strategy “miRNA-seq” and one of the TaxIDs for fungi (txid4751), plants (txid33090) and animals (txid33208). Reference genomes were sourced from the NCBI using the datasets tool, using metadata to calculate global metrics. We focused our strategy on a small selection of samples, including several species with 3 or more distinct projects (*Bocin, Artha, Drmel, Orsat, Caele*). All bioprojects and libraries associated with this project are shown in Table S1. Phylogenetic classifications between sample organisms are derived from NCBI taxonomy.

Assessing sRNA annotator citations was done using NCBI esearch in the pubmed database for publications related to the small RNA annotation tool. This search was performed in April of 2024. Citation counts were compiled from esearch, combining related publications into families (Table S2).

### 5.2 Processing and aligning libraries

The YASMA suite contains several convenience wrappers for managing all processing and alignment steps for the test libraries (Figure S4). These are largely meant to ensure uniformity processing for users. Libraries were downloaded using prefetch and fasterq-dump through the NCBI SRA toolkit (YASMA-download). YASMA-adapter was used to identify common 3’ adapter sequences found in the libraries, which were then trimmed using cutadapt [31] with the following settings `cutadapt -a [3p_adapter_sequence] --minimum-length 15 --maximum-length 50 -O 4 --max-n 0 --trimmed-only [fasta]`. Trimmed libraries were aligned to the reference genome (Table S3) using YASMA-align, which uses bowtie1 [22] and follows the unique-weighting strategy for multi-mapping reads used in ShortStack3/4 and described in [19]. In short, this aligns reads with up to one mismatch, and places multi-mappers with a weighted-random selection based on the number of nearby uniquely mapping reads.

### 5.3 Small RNA annotations

Annotations were done using several approaches. Annotations for ShortStack3 and 4 were done using the same alignments as above, using versions 3.8.5 and 4.0.2, respectively. Annotations using YTO are described here in detail.

To build an alignment depth profile across the genome, YTO performs sequential smoothing steps to reduce the influence of noisy, sparse, and uneven expression on locus identification. First, Genomic coverage is calculated based on the sum of all read counts within a user-defined sliding window (default: 250 nucleotides) centered on each genomic position. To further smooth the profile, YTO performs “padding” by calculating the maximum coverage within a window surrounding position as before, again using a user-defined defined sliding window (default: 100 nucleotides). Padded coverages are normalized to reads-per-aligned-billion (RPB), which are then averaged between replicates by median and averaged between experimental conditions by mean. Condition averaging is not applicable in this work, as we tested only a single condition each of the bioprojects. Annotation read- and genome-percentage tradeoffs are then calculated based on the condition-averaged coverages.

YTO balances the inverse relationship of annotated reads with annotated genomic space, finding a threshold value (in RPB) which balances each. This method follows the approach outlined in kneedle [35] and is summarized in Fig. S10. First, YTO scales the genomic space percentage (genome_scaling_factor = 0.4), reducing the influence of smaller and denser genomes on the threshold (Fig. S10A). Knee discovery involves mapping our scaled genomic space vs the percent of reads annotated as a curve, with precision up to 4 decimals. We identify perpendicular and vertical distance (Pdist and Vdist) for this curve against an x = y line (Fig. S10B), calculating the point of maximum Pdist-Vdist as the threshold or “knee” (Fig. S10C).

YTO then identifies candidate regions based on positions which exceed the RPB threshold. Regions are expanded and/or trimmed to define the boundary where expression is largely lost (<5 % of the expression at the edge). Regions are merged sequentially if they meet all the following standards: 1) they must have overlapping size profiles or have no-predominant size, 2) they must have a similar proportion of reads that come from the top strand (a difference in proportion no greater than 0.5 by default), and 3) they must be close enough (less than 500 nucleotides by default). Loci are merged greedily by this method, with recalculation of characteristics for the previously unannotated area between merged regions.

Loci are finally subjected to filters based on three criteria. First, we apply a minimum abundance threshold (default: 50 reads), ensuring that all annotated loci are of sufficient abundance to give an accurate portrayal of their sRNA-size profile and stranding. This also avoids cases where sequencing noise might be annotated as a locus in low-abundance libraries. Second, we apply a minimum abundance density threshold (default: 100 reads / kBase), aimed at catching any large loci which might be the result of over-merging loci produced from noise. Third, we apply a minimum complexity filter (default: 10 unique reads / kBase). All known sRNA loci produce multiple sequences, both from distinct sequences and related sequence variants. YTO considers this a hallmark of a real locus, expecting that sufficiently deep loci will contain a number of unique sequences relative to their genomic size. This filter largely overlaps with the prior two criteria but can also find loci which might be the result of technical errors like sequence duplication, which is challenging to filter in sRNA-sequencing. This minimum standard is intended to retain miRNA loci, requiring that they would contain 2 sequences for a 200 nucleotide hairpin, possibly coming from processing variants or a miRNA-star. Together, these filters ensure loci are deep and complex enough to be usefully analyzed and reduce the chances of annotating noise as loci. In general, these filters favor annotative precision over sensitivity, considering false positives to be a substantial risk. While each of these abundance filters can be modified or removed, the recommended remedy is to sequence with greater library depth.

### 5.4 Genomic intersections

Intersections and comparisons of loci were performed using the GenomicRanges package in R [23]. Jaccard index was calculated between annotations using the following formula: $\frac{\mathrm{Annotation\_intersection}}{\mathrm{Annotation\_union}}$

Simple matching coefficients were calculated using the following formula: $\frac{\mathrm{Annotation\_intersection}+\mathrm{Unannotated}}{\mathrm{Genome\_length}}$.

## Author contributions

NRJ conceived of the project. NRJ designed and implemented YASMA. NRJ and FGT performed test annotations, analysis, and troubleshooting of the YASMA methodology. BBG performed the analysis of available annotators. FGT and BBG searched and annotated the metadata of test projects and libraries.

## CRediT authorship contribution statement

**Johnson Nathan:** Writing – review & editing, Writing – original draft, Visualization, Validation, Supervision, Software, Resources, Project administration, Methodology, Investigation, Funding acquisition, Formal analysis, Data curation, Conceptualization. **Fabian Gonzalez-Toro:** Writing – review & editing, Validation, Data curation, Conceptualization. **Barbara Bernal Gomez:** Data curation.

## Funding

This work was funded by the National Agency for Research and Development of Chile (ANID) FONDECYT program (11220727) awarded to NRJ. NRJ also recieved support from the ANID–Millennium Science Initiative Program–Millennium Institute for Integrative Biology (ICN17_022).

## Declaration of Competing Interest

I declare that this work is original to our laboratory and authors and has not been published elsewhere, save for a preprint submission (bioRxiv). We have no conflicts of interest to declare with this work and we have no financial, personal, or professional conflicts that otherwise might affect the integrity of this work. Our work is funded by the National Agency for Research and Development (ANID) of Chile through a Fondecyt (11220727) grant and their Millennium Institute for Integrative Biology (iBio).

## Data Availability

All sequencing data for this study are available in the NCBI-SRA and are outlined in Table S2. All genomes used for alignments are from the NCBI genome database and are enumerated in Table S3. The YASMA annotation suite is available at https://github.com/NateyJay/YASMA under the GPL-3.0 license. Installation is performed by simply running the yasma.py script from the github repository folder, with more explicit instructions available on its page. YASMA has been tested in Mac-OS and Linux systems and is written for Python3. YTO annotation can be used on any bam-formatted sRNA alignment, only additionally requiring the RG:Z samtools tag which indicates the library source for library-specific scaling.
