# Supplementary material for: Class-agnostic annotation of small RNAs balances sensitivity and specificity in diverse organisms
Source: Comput Struct Biotechnol J. 2025 May 27;27:2450–9. doi: 10.1016/j.csbj.2025.05.045 (PMC12174571; doi:10.1016/j.csbj.2025.05.045)
Supplement: Supplementary file 1 — Supplementary material [file mmc1.docx]

# Supplemental materials:


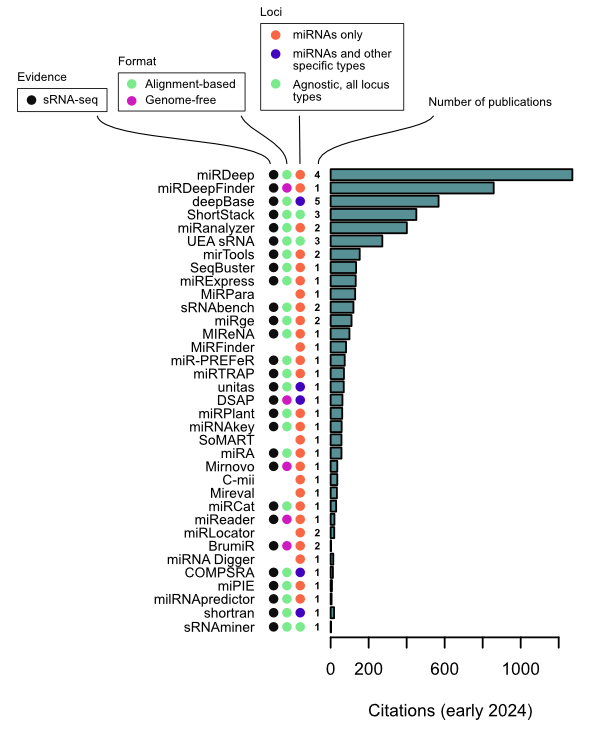


#### Figure S1 - annotator overview

Overview of published sRNA annotators. Tools with multiple publications and versions are aggregated by citation count, listing the number of publications containing this information (Table S1). Tools are described in terms of their scope and strategy, first identifying which are sRNA-seq based. Format of annotation and what locus-types are described are also shown with the described colors.


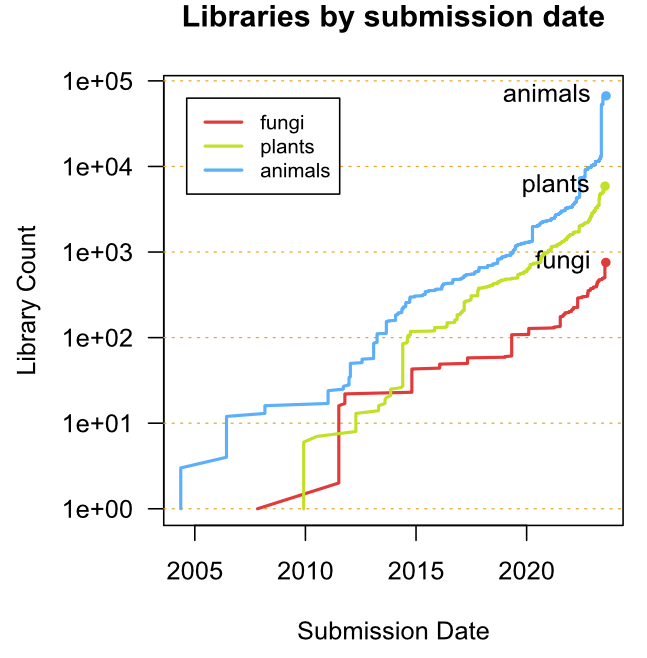


#### Figure S2 - NCBI assessment

Cumulative count of “miRNA-seq” libraries in the NCBI-SRA, aggregated by sample kingdom.


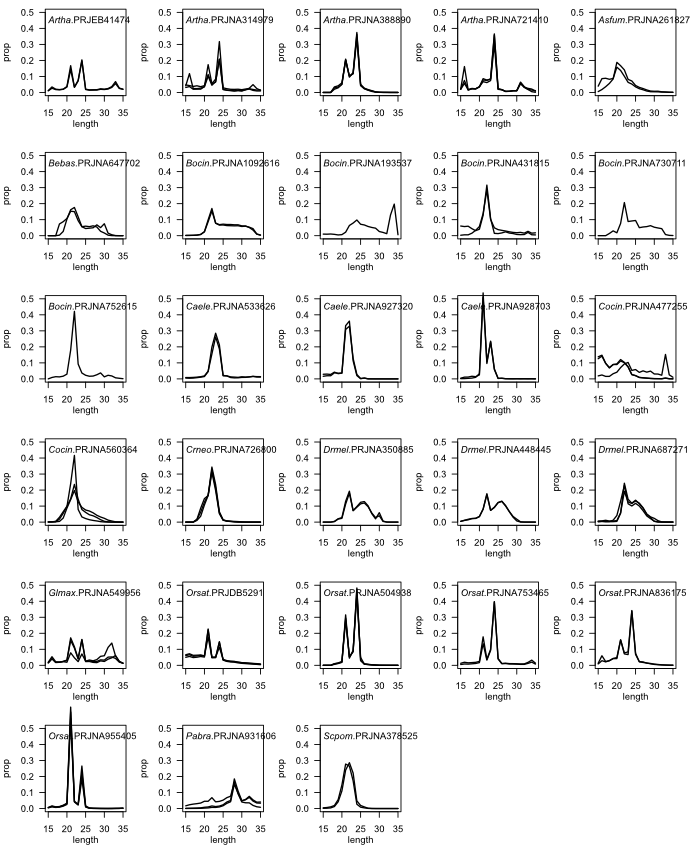


#### Figure S3 - Size profiles of aligned sRNAs

Size profile of all projects included in this analysis. Profiles are proportions of all aligned reads, with libraries shown as different overplotted lines.


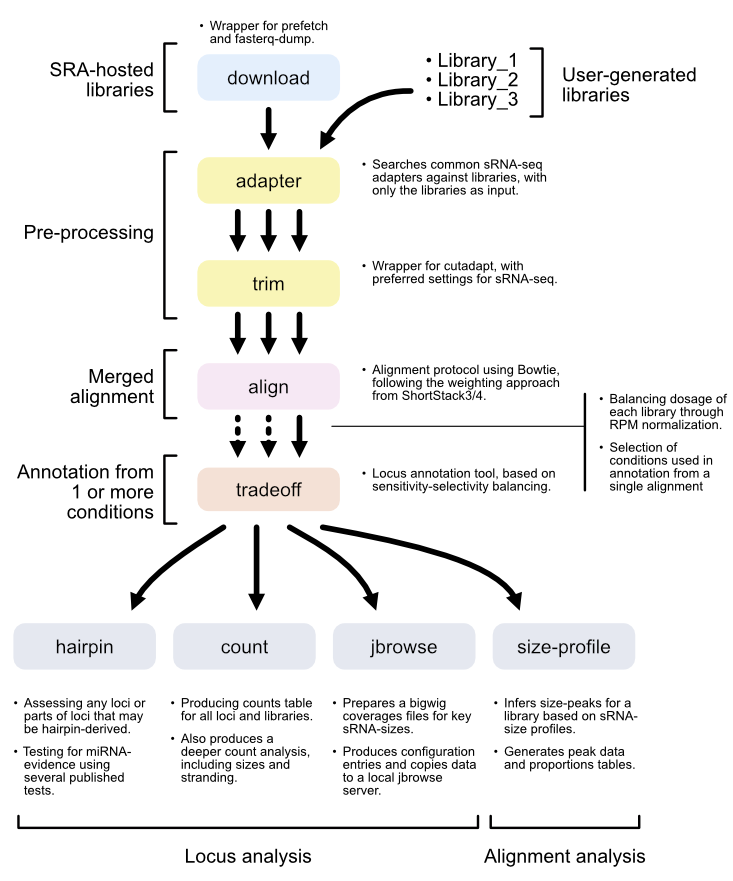


#### Figure S4 - Overview of YASMA suite

Block diagram indicating the modules and general analysis pipeline for YASMA. YASMA-tradeoff is the focus of this publication.


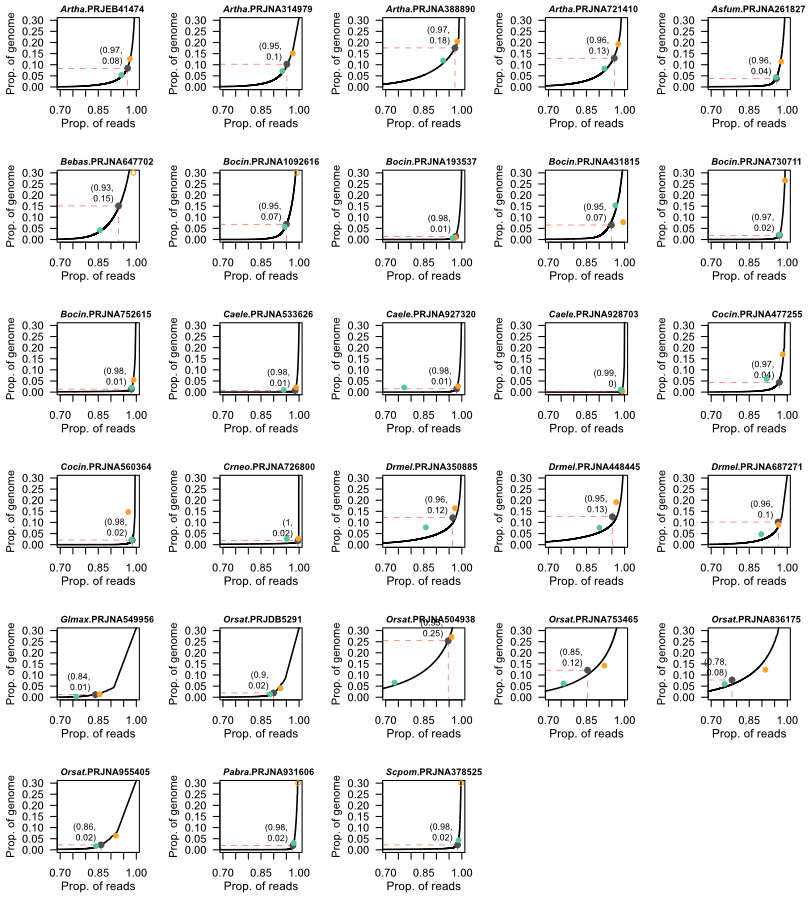


#### Figure S5 - tradeoff curves

Tradeoff curves of the proportion of genome annotated vs the proportion of reads annotated for each project. Curves are calculated by YASMA-tradeoff (YTO), with the final annotation rates reported for each tool: YTO (black), ShortStack3 (orange), and ShortStack4 (aquamarine).


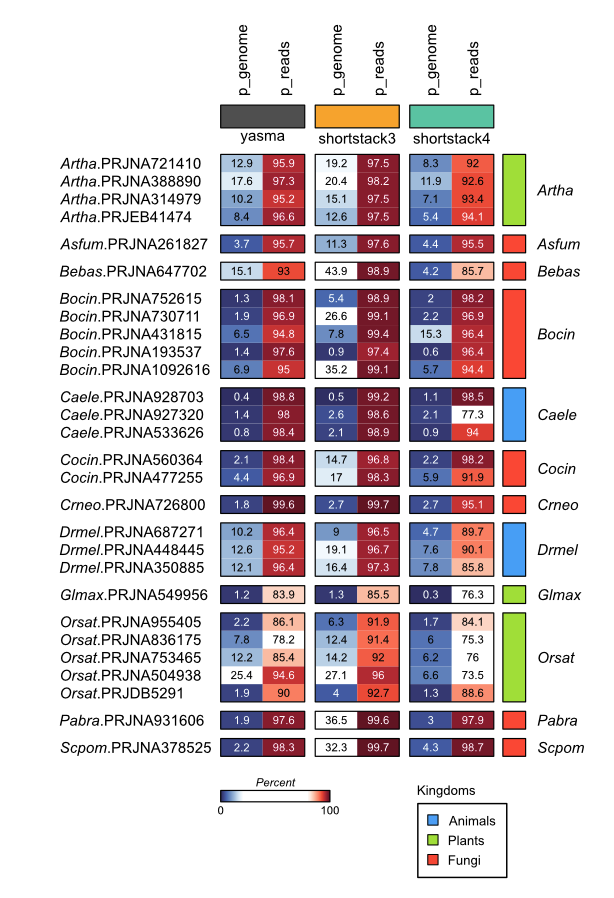


#### Figure S6 - annotation rates for all tools and projects

A summarization of figures 2C and S5, showing the read and genome annotation rates for each annotation. Kingdoms are identified by colors: animals (light blue), plants (light green), and fungi (bright red). Percents are shown as color scales, with blues shown from 0-20%, white for 20-80%, and reds for 80-100%.


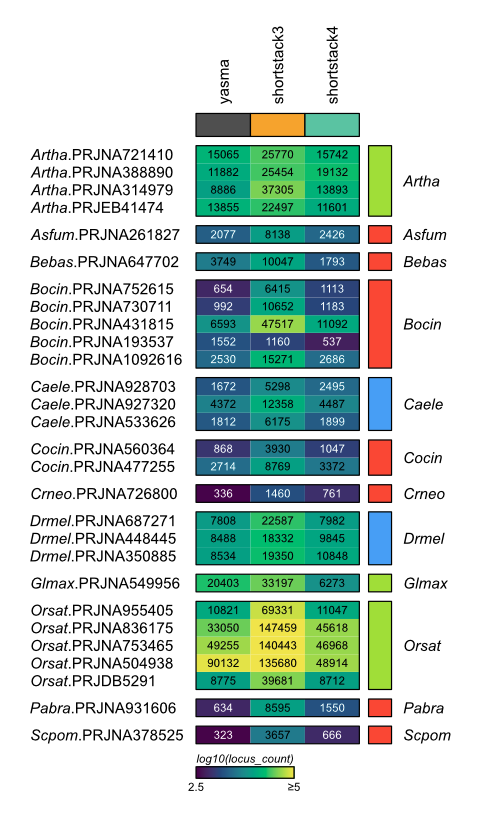


#### Figure S7 - locus counts

Heatmap of locus counts for each project and annotator. Kingdoms are colored on the right as plants (light green), animals (light blue), and fungi (bright red).


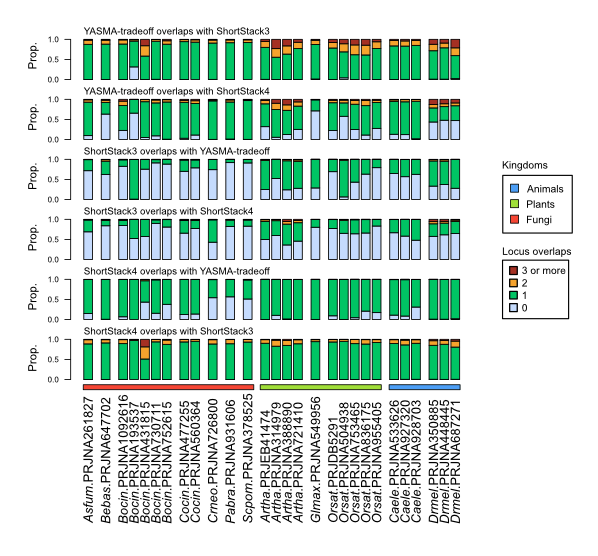


#### Figure S8 - locus overlap between annotations

An extension of figure 3E, showing pairwise overlaps for all annotations.


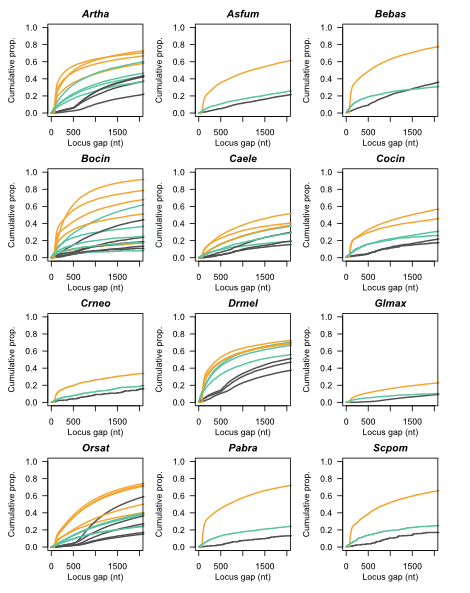


#### Figure S9 - Inter-locus gaps for annotations

Inter-locus gap distances are represented as a cumulative density function for each annotator, aggregated by organism. Lines represent specific annotations and line color indicates the annotator: YASMA-tradeoff (black), ShortStack3 (orange), and ShortStack4 (aquamarine).


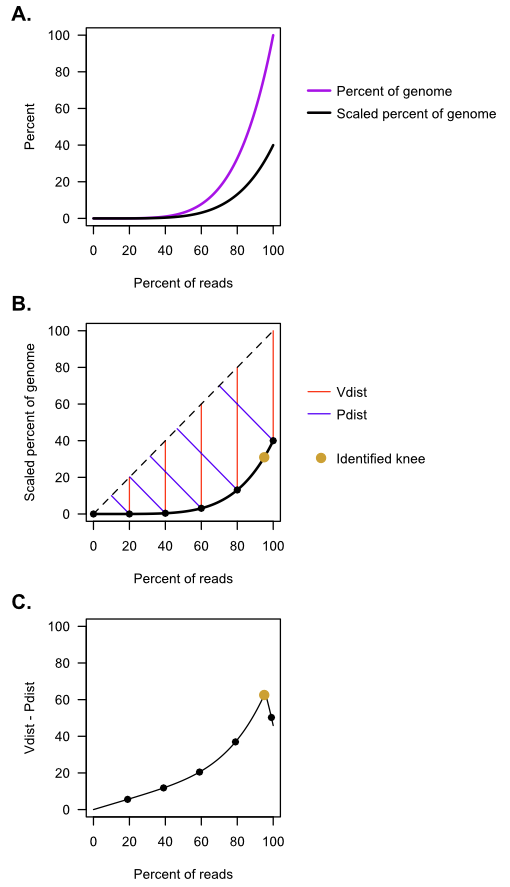


#### Figure S10 – Knee discovery example

An example of the Knee-finding algorithm with toy data.

1. Tradeoff curve, showing the default scaling for genome annotation percentage.
2. Calculations for vertical and perpendicular distance, shown for 6 points.
3. Calculated knee score, indicating the identified knee.

#### Table S1 - Table of tool publications

#### Table S2 - Table of libraries

#### Table S3 - Table of genomes
